# Bone and Joint Tissue Penetration of the *Staphylococcus*-Selective Antibiotic Afabicin in Patients Undergoing Elective Hip Replacement Surgery

**DOI:** 10.1128/AAC.01669-18

**Published:** 2019-02-26

**Authors:** Annick Menetrey, Annick Janin, John Pullman, J. Scott Overcash, Amina Haouala, François Leylavergne, Laurent Turbe, Frederick Wittke, Valérie Nicolas-Métral

**Affiliations:** a Debiopharm International SA, Lausanne, Switzerland; b St. James Healthcare, SCL Health, Butte, Montana, USA; c Sharp Grossmont Hospital, La Mesa, California, USA; d Atlanbio, Saint-Nazaire, France

**Keywords:** Debio 1450, *Staphylococcus aureus*, afabicin, drug development, drug penetration, joint infections, osteomyelitis, pharmacokinetics

## Abstract

Afabicin (formerly Debio 1450, AFN-1720) is a prodrug of afabicin desphosphono (Debio 1452, AFN-1252), a novel antibiotic in development which targets the staphylococcal enoyl-acyl carrier protein reductase (FabI) and exhibits selective potent antibacterial activity against staphylococcal species, including methicillin-resistant Staphylococcus aureus. As part of clinical development in bone and joint infections, a distribution study in bone was performed in 17 patients who underwent elective hip replacement surgery. Patients received 3 doses of 240 mg afabicin orally (every 12 h) at various time points before surgery. Afabicin desphosphono concentrations were measured by liquid chromatography-tandem mass spectrometry in plasma, cortical bone, cancellous bone, bone marrow, soft tissue, and synovial fluid collected during surgery at 2, 4, 6, or 12 h after the third afabicin dose. The study showed good penetration of afabicin desphosphono into bone tissues, with mean area under the curve ratios for cortical bone-, cancellous bone-, bone marrow-, soft tissue-, and synovial fluid-to-total plasma concentrations of 0.21, 0.40, 0.32, 0.35, and 0.61, respectively. When accounting for the free fraction in plasma (2%) and synovial fluid (9.4%), the mean ratio was 2.88, which is indicative of excellent penetration and which showed that the afabicin desphosphono concentration was beyond the MIC_90_ of S. aureus over the complete dosing interval. These findings, along with preclinical efficacy data, clinical efficacy data for skin and soft tissue staphylococcal infection, the availability of both intravenous and oral formulations, and potential advantages over broad-spectrum antibiotics for the treatment of staphylococcal bone or joint infections, support the clinical development of afabicin for bone and joint infections. (This study has been registered at ClinicalTrials.gov under identifier NCT02726438.)

## Abstract (precis)

Afabicin (formerly Debio 1450, AFN-1720) is a prodrug of afabicin desphosphono (Debio 1452, AFN-1252), a novel antibiotic in development which targets the staphylococcal enoyl-acyl carrier protein reductase (FabI) and exhibits selective potent antibacterial activity against staphylococcal species, including methicillin-resistant Staphylococcus aureus. As part of clinical development in bone and joint infections, a distribution study in bone was performed in 17 patients who underwent elective hip replacement surgery.

## INTRODUCTION

The treatment of bone and joint infections remains difficult, usually involving a prolonged course of antibiotics, often with surgical intervention, due to the poor vascularization at the site of infection. Consequently, the efficacy of an antimicrobial to treat such infections depends on its ability to penetrate these compartments, in addition to its activity against the underlying pathogen (1). Staphylococci, including methicillin-resistant Staphylococcus aureus (MRSA), are the predominant identified causative pathogens of these infections; the prevalence of S. aureus as the cause of infection ranged from 30% to 60% for osteomyelitis cases and 39% to 76% for septic arthritis cases, dependent on differences between acute and chronic infections, study country, and anatomic location (2–4). These numbers highlight the large medical and economic burden of S. aureus as a causative pathogen in osteoarticular infections.

Current treatment guidelines recommend the use of broad-spectrum antibiotics, in addition to surgical intervention for debridement of devitalized bone or removal of an infected prosthetic device for both culture and successful healing (5). Continued use of broad-spectrum antibiotics is indicated unless bone or joint fluid cultures allow for more focused and selective antibiotic therapy. Their use has been implicated in the disturbance of the commensal gut microbiota, leading to the spread of antibiotic resistance and increased colonization by various gut pathogens, such as Clostridium difficile and Salmonella enterica serovar Typhimurium (6–8). Furthermore, despite the availability of these broad-spectrum antibiotics and advances in diagnostic and surgical techniques, osteoarticular infections continue to be associated with significant morbidity and mortality. Septic arthritis is considered a medical and surgical emergency, associated with a mortality rate of about 11% (9). Ten to 30% of patients with septic arthritis suffer long-term decreased joint function or mobility (4). Both acute and chronic osteomyelitis results in inflammatory bone destruction, bone necrosis, and new bone formation. The short-term mortality rates for osteomyelitis are 2.8 to 7.7% for nonvertebral osteomyelitis and 6 to 16% for vertebral osteomyelitis (4). The mortality rate due to prosthetic joint infection (PJI) caused by S. aureus has been reported to be between 0% and 7% (5).

Afabicin (formerly Debio 1450, AFN-1720), a prodrug of afabicin desphosphono (Debio 1452, AFN-1252), belongs to a new class of antibiotic that targets bacterial fatty acid biosynthesis by inhibiting the enoyl-acyl carrier protein reductase (FabI). Afabicin desphosphono exhibits selective antibacterial activity against both coagulase-negative and -positive staphylococci, including MRSA, and can be administered intravenously and orally. The MIC_90_ against recent MRSA isolates (collected in 2015 and 2016) is 0.008 µg/ml, with 99.4% of organisms being inhibited at a concentration of 0.06 µg/ml (10). Afabicin desphosphono does not show cross-resistance with other antibacterial classes typically used to treat infections caused by Gram-positive pathogens (10).

The *in vivo* efficacy of afabicin has been demonstrated in multiple animal models of staphylococcal infection, including models of osteomyelitis, where it showed significant activity and high bone-to-plasma ratios of its active moiety (11, 12). Furthermore, afabicin desphosphono showed the potential to eradicate intracellular S. aureus in osteoblasts (13). The efficacy of afabicin was also demonstrated in the clinical setting in a phase 2 study in patients with acute bacterial skin and skin structure infection (ABSSSI), where treatment involved a switch from the intravenous (i.v.) to the oral route. Afabicin treatment was noninferior to the comparator, with an overall good safety and tolerability profile (unpublished data). Finally, the effect of a 20-day oral afabicin administration on the human gut microbiota was assessed in 15 healthy volunteers: no significant changes were observed, supporting the premise that targeted antibiotherapy to treat staphylococcal infections may reduce antibiotic-associated complications, such as antibiotic-associated diarrhea and C. difficile infections (14).

The narrow-spectrum activity of afabicin, its efficacy in an animal osteomyelitis model, its availability as both i.v. and oral formulations, as well as its promising bone penetration in drug distribution studies in animals suggest that it might be a valuable innovative therapeutic option for the treatment of staphylococcal osteoarticular infections. With adequate human tissue exposure in sites of infection being a key driver of efficacy, a phase 1 study was conducted in patients undergoing elective hip replacement surgery to evaluate the pharmacokinetics of afabicin in human bone and articular tissue and its potential for the treatment of staphylococcal bone and joint infections.

## RESULTS

Seventeen patients were enrolled. The mean age for the 15 patients dosed with afabicin was 59.7 years (range, 37 to 75 years); 53% of subjects were males. The mean body mass index (BMI) was 30.2 kg/m^2^ (range, 24 to 35 kg/m^2^).

Among the 15 patients who received afabicin during the study, afabicin was generally well tolerated. There were 3 serious adverse events (SAEs) reported by 3 patients (moderate muscle spasms, moderate paralytic ileus, and moderate pneumonia); none were considered to be related to afabicin. There were no adverse events (AEs) leading to death. One patient (6.7%) discontinued the study drug due to moderate vomiting and severe presyncope, which were considered related to afabicin dosing; these events resolved spontaneously. For this patient, no samples were available for pharmacokinetic (PK) assessments. Among the five patients who had postdose electrocardiographic (ECG) assessments, two patients experienced AEs of mild QT prolongation considered related to afabicin; these AEs resolved on follow-up ECGs. One patient out of 15 (6.7%) experienced AEs of a mild alanine aminotransferase (ALT) increase, a mild aspartate aminotransferase (AST) increase, and a mild gamma-glutamyltransferase (GGT) increase, and all events were considered related to afabicin; all 3 events were resolved or were resolving on follow-up testing.

### Plasma pharmacokinetic concentrations and parameters.

**FIG 1:**
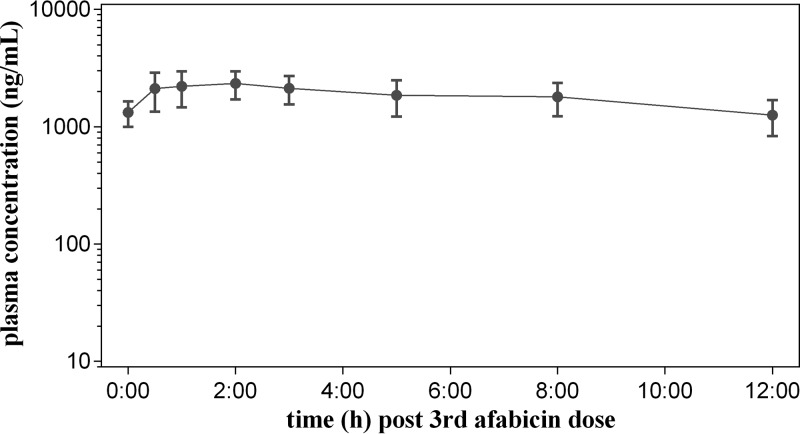
Profile of the afabicin desphosphono concentration in plasma after the third oral dose of afabicin (240 mg, q12h) in patients undergoing hip replacement surgery. The results are presented as the geometric mean ± SD obtained using data from nominal time points (number of patients = 14).

Only 14 patients out of 15 who received afabicin completed the study and were included in the PK population. The mean ± standard deviation (SD) afabicin desphosphono concentrations in plasma are presented in Fig. 1. Following the third presurgical dose of afabicin (every 12 h [q12h]), 3 of 14 patients had afabicin plasma concentrations above the limit of quantification (<5 ng/ml) at 0.5 h postdose; then, all results were below the limit of quantification at 1 h through 12 h postdose. Following oral administration, the prodrug afabicin is rapidly converted to the active moiety, afabicin desphosphono. Since afabicin is pharmacologically inactive and almost absent from the systemic circulation, the concentrations of afabicin desphosphono are relevant only for evaluation of drug exposure and penetration in tissues. The pharmacokinetic parameters for afabicin desphosphono are summarized in Table 1. Peak concentrations were observed at approximately 2 h postdose (mean, 2,360 ng/ml; coefficient of variation [CV], 28.0%) and declined thereafter in a monoexponential manner with a mean half-life (*t*_1/2_) of 11.8 h (CV, 70.1%). At 12 h postdose, the concentration (mean, 1,120 ng/ml; CV, 43.4%) was comparable to the predose concentration (mean, 1,200 ng/ml; CV, 36.7%), indicating that the afabicin desphosphono steady state was reached after the third dose of afabicin. The mean area under the curve (AUC) over the dosing interval (12 h) at steady state (AUC_τ_) was 20,400 ng·h/ml (CV, 30.6%), consistent with the range observed in previous studies of healthy volunteers.

**TABLE 1:** PK parameters of afabicin desphosphono in plasma and bone tissues and fluid after the third oral dose of afabicin in patients undergoing hip replacement surgery^*d*^

Parameter	Value(s) for:						
	Plasma (*n* = 14)	Sparse sampling (from *n* = 2–4 at each time of resection)					
		Plasma	Cortical bone	Cancellous bone	Bone marrow	Soft tissue	Synovial fluid
AUC_τ_ (ng·h/ml for fluids or ng·h/g for tissues)	20,400 (30.6)	20,100	4,240	8,040	7,060	6,790	12,300
*C*_12_ (ng/ml for fluids or ng/mg for tissues)	1,120 (43.4)	1,500	373	648	640	526	1,210
*C*_max_ (ng/ml for fluids or ng/mg for tissues)	2,360 (28.0)	2,150	441	841	695	759	1,280
C_trough_^*a*^ (ng/ml for fluids or ng/mg for tissues)	1,200 (36.7)	—	—	—	—	—	—
*t*_1/2_^*b*^ (h)	11.8 (70.1)	—	—	—	—	—	—
*t*_max_ (median [range]) (h)	2 (0.5–2)	6	6	6	6	6	6
Penetration ratio							
AUC for tissue/AUC for plasma	—	1	0.21	0.40	0.35	0.34	0.61
*C*_max_ for tissue/*C*_max_ for plasma	—	1	0.21	0.39	0.32	0.35	0.60
AUC for free tissue/AUC for free plasma^*c*^	—	1	—	—	—	—	2.88

a *C*_trough_ was not obtained for bone resection-associated sparse sampling, as surgery occurred after the 3rd afabicin dose.

b *t*_1/2_ was not calculated for data obtained from sparse samples, as an elimination phase could not be characterized from the mean profile.

c The free fraction of afabicin desphosphono was 2% in plasma and 9.4% in synovial fluid.

d Afabicin was administered at 240 mg q12h. Results are geometric means (geometric CV [in percent]) unless otherwise indicated. AUC_τ_, area under the curve at steady state; *C*_12_, concentration 12 h after the last dose; *C*_max_, maximum observed plasma concentration; *C*_trough_, measured concentration at the end of the dosing interval at steady state, just prior to the last dose; *t*_1/2_, terminal elimination half-life; *t*_max_, time of maximum observed plasma concentration; *n*, number of observations; —, no reportable data.

### Tissue concentrations and pharmacokinetic parameters.

A method for quantification of afabicin desphosphono in human bone homogenates was successfully developed and validated. However, a difference (20 to 25%) was observed between the concentrations of the replicates of the first samples tested, probably due to inhomogeneity between the aliquots from the collected samples. Consequently, in order to increase the accuracy of the measurement, analyses of at least duplicates of aliquots were performed for each tissue. If the difference between the duplicates was higher than 25% or if the difference between the tissues was higher than 50%, repeat analyses were performed in duplicate. The median for all replicates was reported as recommended by the Global Bioanalysis Consortium Harmonization Team (15). Detailed results are presented in Table S1 in the supplemental material.

**FIG 2:**
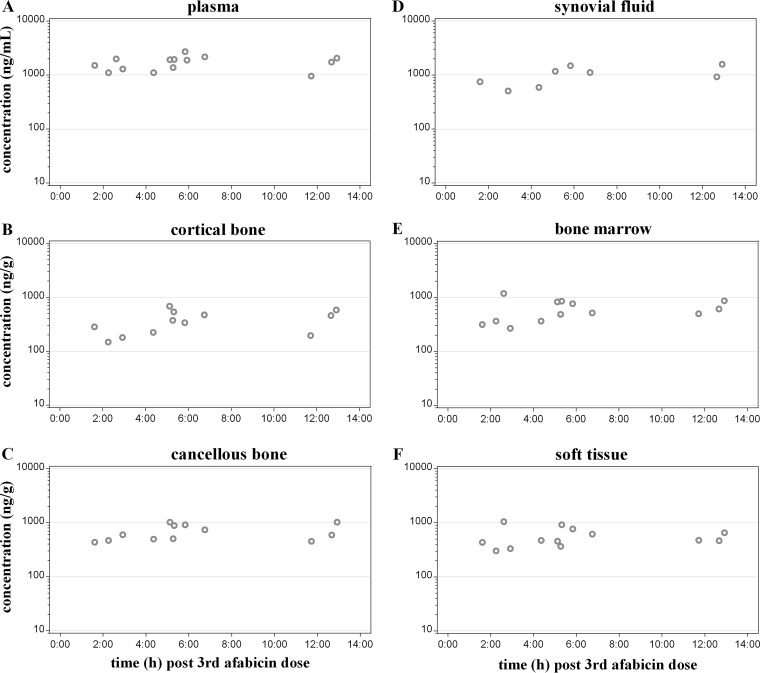
Individual afabicin desphosphono concentrations in plasma (A), cortical bone (B), cancellous bone (C), synovial fluid (D), bone marrow (E), and soft tissue (F) after the third oral dose of afabicin (240 mg, q12h) in patients undergoing hip replacement surgery. Data are from 14 patients, with the following exceptions: for 1 patient, the plasma sample was missing at the time of resection and the plasma concentration was extrapolated by linear regression between the two adjacent concentration data from the full PK profile in plasma. Synovial fluid was obtained for 8 patients only. For one patient no results except for plasma were reportable at the time of resection, and for another, only plasma, soft tissue, and bone marrow were collected at the time of resection.

The concentrations of afabicin desphosphono measured at the time of resection were the highest at the 6-h postdose time point for each tissue or fluid (plasma, cortical bone, cancellous bone, bone marrow, soft tissue, and synovial fluid) and were still measurable at 12 h postdose in each tissue or fluid (Table 2; Fig. 2).

**TABLE 2:** Concentrations of afabicin desphosphono at time of resection after the third oral dose of afabicin in patients undergoing hip replacement surgery^*f*^

Compartment	Concn at:				
	2 h after 3rd dose (*n* = 4)	4 h after 3rd dose (*n* = 3)	6 h after 3rd dose (*n* = 4)	12 h after 3rd dose (*n* = 3)	Mean (*n* = 14)
Plasma (ng/ml)	1,430 (25.4)	1,420 (28.3)	2,150 (16.5)	1,500 (41.8)	1,620 (31.0)
Cortical bone (ng/g)	196 (34.2)^*b*^	385 (60.5)	441 (24.3)^*b*^	373 (62.5)	334 (53.8)^*d*^
Cancellous bone (ng/g)	496 (16.9)^*b*^	635 (42.8)	841 (11.4)^*b*^	648 (43.2)	644 (33.7)^*d*^
Bone marrow (ng/g)	436 (76.1)	526 (43.5)	695 (27.0)^*b*^	640 (28.3)	554 (47.8)^*e*^
Soft tissue (ng/g)	464 (61.5)	430 (13.8)	759 (20.3)^*b*^	526 (19.2)	525 (39.6)^*e*^
Synovial fluid (ng/ml)	614 (28.5)^*a*^	829 (51.7)^*a*^	1,280 (20.6)^*a*^	1,210 (39.2)^*a*^	943 (43.5)^*c*^

a Data are for 2 observations.

b Data are for 3 observations.

c Data are for 8 observations.

d Data are for 12 observations.

e Data are for 13 observations.

f Afabicin was administered at 240 mg q12h. Results are geometric means (geometric CV [in percent]) by time point and overall by tissue or fluid; *n*, number of patients. Unless indicated otherwise in a footnote, the data are for a number of observations equal to the number of patients.

The mean concentrations of afabicin desphosphono between the 2- and 12-h time points ranged from 196 to 441 ng/g in cortical bone, from 496 to 841 ng/g in cancellous bone, from 436 to 695 ng/g in bone marrow, from 430 to 759 ng/g in soft tissue, and from 614 to 1,280 ng/ml in the synovial fluid.

Pharmacokinetic parameters estimated from the sparse afabicin desphosphono concentrations measured during bone resection are presented in Table 1. The exposure derived from the composite profile constructed from the sparse plasma samples at the time of resection (AUC_τ_, 20,100 ng·h/ml; maximum observed plasma concentration [*C*_max_], 2,150 ng/ml) was similar to that measured from the serial PK samples (mean AUC_τ_, 20,400 ng·h/ml; *C*_max_, 2,360 ng/ml; the values were averaged from the individual values derived for each full PK plasma profile), even if the mean profile derived from sparse concentrations differed (notably, the composite time to *C*_max_ [*t*_max_; 6 h], which was different from the median *t*_max_ [2 h] for individual values) due to the limited number of values at each time point (*n* = 2 to 4) compared to the rich full PK plasma data (*n* = 14). The fact that plasma AUCs were similar demonstrates the adequacy of deriving an AUC value for tissues based on the composite profile. Exposures in bone marrow, cancellous bone, and soft tissue were comparable (AUC_τ_ range, 7,060 to 8,040 ng·h/g; *C*_max_ 695 to 841 ng/g), while the exposure in cortical bone (AUC_τ_, 4,240 ng·h/g; *C*_max_, 441 ng/g) was the lowest among the bone tissues. The exposure of afabicin desphosphono in the synovial fluid (AUC_τ_, 12,300 ng·h/ml; *C*_max_, 1,280 ng/ml) was the highest of all the bone tissues. The median *t*_max_ was 6 h in each tissue.

### Tissue-to-plasma ratios.

The ratios of afabicin desphosphono measured in each type of bone tissue and fluid versus the plasma concentration at the time of bone resection remained constant over time from 2 to 12 h postdose (Table 3). Mean cortical bone-, cancellous bone-, bone marrow-, soft tissue-, and synovial fluid-to-plasma ratios obtained overall between the 2- to 12-h time points in this study were 0.21, 0.41, 0.35, 0.33, and 0.54, respectively. This was consistent with the mean AUC_τ_ ratios for cortical bone-, cancellous bone-, bone marrow-, soft tissue-, and synovial fluid-to-total plasma concentrations of 0.21, 0.40, 0.35, 0.34, and 0.61, respectively (Table 1). Individual ratios to plasma concentrations ranged from 0.13 to 0.36 for cortical bone, from 0.29 to 0.53 for cancellous bone, from 0.21 to 0.60 for bone marrow, from 0.24 to 0.53 for soft tissue, and from 0.39 to 0.77 for synovial fluid. The ratios obtained for cortical bones and synovial fluid were the lowest and highest among the bone tissues, respectively.

**TABLE 3:** Bone tissue- and fluid-to-plasma concentration ratios of afabicin desphosphono at the time of resection after the third oral dose of afabicin in patients undergoing hip replacement surgery^*f*^

Compartment	Concn at:				
	2 h after 3rd dose (*n* = 4)	4 h after 3rd dose (*n* = 3)	6 h after 3rd dose (*n* = 4)	12 h after 3rd dose (*n* = 3)	Mean (*n* = 14)
Cortical bone (ng/g)	0.15 (18.6)^*b*^	0.27 (28.7)	0.20 (42.7)^*b*^	0.25 (17.4)	0.21 (34.2)^*d*^
Cancellous bone (ng/g)	0.39 (25.4)^*b*^	0.45 (18.7)	0.38 (17.0)^*b*^	0.43 (20.9)	0.41 (19.3)^*d*^
Bone marrow (ng/g)	0.30 (53.1)	0.37 (14.0)	0.31 (32.4)^*b*^	0.43 (20.0)	0.35 (34.0)^*e*^
Soft tissue (ng/g)	0.32 (34.2)	0.30 (31.9)	0.34 (30.0)^*b*^	0.35 (32.4)	0.33 (28.5)^*e*^
Synovial fluid (ng/ml)	0.44 (17.4)^*a*^	0.57 (9.6)^*a*^	0.53 (4.5)^*a*^	0.64 (26.3)^*a*^	0.54 (19.3)^*c*^

a Data are for 2 observations.

b Data are for 3 observations.

c Data are for 8 observations.

d Data are for 12 observations.

e Data are for 13 observations.

f Afabicin was administered at 240 mg q12h. Results are geometric means (geometric CV [in percent]) by time point and overall by tissue or fluid; *n*, number of patients. Unless indicated otherwise in a footnote, the data are for a number of observations equal to the number of patients.

### Protein binding in plasma and synovial fluid.

The afabicin desphosphono free fraction was 2% in blank plasma samples spiked with afabicin desphosphono as well as in plasma samples collected from the patients during the study. This confirmed the values obtained in previous *in vitro* and *in vivo* experiments (data not shown). Individual protein binding in synovial fluid collected during the study could not be determined due to a limited sample volume. However, protein binding was determined in synovial fluids obtained from different sources after spiking with afabicin desphosphono: individual free fractions ranged from 2.4% to 30.4%, with a mean value of 9.4% and a median value of 5.4%. Considering these free fractions, the mean ratio of the unbound AUC for synovial fluid tissue over the unbound AUC for plasma was 2.88 (Table 1).

## DISCUSSION

The most common Gram-positive pathogen associated with osteomyelitis is S. aureus. For acute hematogenous osteomyelitis and septic arthritis, the typical treatment duration is 4 to 6 weeks; for chronic osteomyelitis, it is 8 to 12 weeks, with recent treatment guidelines suggesting 6 to 10 weeks (16, 17). Afabicin belongs to a new class of antibiotic which targets the enoyl-acyl carrier protein reductase (FabI), which plays a key role in fatty acid biosynthesis in *Staphylococcus*. Its active moiety exhibits selective potent antibacterial activity against *Staphylococcus* (10), and afabicin was recently shown to be effective in patients with ABSSSI in a phase 2 study (unpublished data). As plasma concentrations may be of limited use in the context of infections in the bone and joint, the purpose of the current study was to measure afabicin desphosphono concentrations at relevant sites of infection, namely, cortical and cancellous bones, bone marrow, soft tissue, and synovial fluid. The 240-mg oral dose of afabicin was generally well tolerated when administered to patients as 3 presurgical oral doses approximately 12 h apart. Plasma concentration data confirmed that afabicin desphosphono reached steady state by the third dose of afabicin. Mean afabicin desphosphono AUC ratios for cortical bone-, cancellous bone-, bone marrow-, soft tissue-, and synovial fluid-to-plasma ratios were 0.21, 0.40, 0.35, 0.34, and 0.61, respectively. The penetration of afabicin desphosphono was the highest for synovial fluid (individual ratios ranged from 0.39 to 0.77). Penetration into bone marrow, soft tissue, and cancellous bone were all similar (individual ratios ranged from 0.21 to 0.60). The lowest penetration was observed for cortical bone, with individual ratios ranging from 0.13 to 0.36. The ratios remained constant over time between 2 and 12 h postdose. Of note, considering that only free drug can penetrate into the tissues, then the total tissue concentrations may more appropriately represent the bioactive drug concentration within the tissues and should be compared to the free concentrations in plasma to evaluate the extent of tissue penetration, as it has been discussed for other antibiotics, such as oritavancin (18) or tigecycline (19). The ratios of the tissue concentrations to the free plasma concentrations of afabicin desphosphono then ranged from 14.5 to 26.7 for cancellous bone, 6.3 to 17.9 for cortical bone, 10.4 to 26.2 for bone marrow, 19.5 to 38.5 for synovial fluid, and 12.0 to 24.9 for soft tissue.

**FIG 3:**
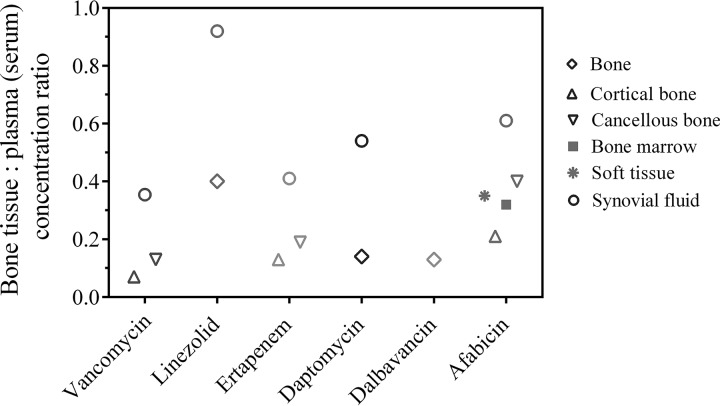
Osteoarticular tissue- and fluid-to-plasma concentration ratios obtained for vancomycin, linezolid, ertapenem, daptomycin, dalbavancin, and afabicin. Mean ratios (median ratios for ertapenem) were obtained from published data from studies with designs similar to that of this study, i.e., dosing in patients undergoing elective replacement surgery and analysis of the total concentration of the drugs in homogenized bone tissues or synovial fluid collected during surgery (20–25) at steady state for linezolid and afabicin and after a single dose for the other antibiotics. Vancomycin, linezolid, and ertapenem were quantified in serum, whereas daptomycin, dalbavancin, and afabicin desphosphono were quantified in plasma.

The total concentrations in tissues allow for a comparison with the reported concentrations of other antibiotics in bone tissues and synovial fluids obtained with a study design similar to that used in the present study, i.e., dosing in patients undergoing elective replacement surgery and analysis of the total concentration of the drug in bone tissues collected during the surgery, after homogenization (Fig. 3). Such tissue concentration-to-plasma/serum concentration ratios at the time of surgery were published notably for vancomycin, linezolid, ertapenem, and daptomycin, and AUC_tissue_-to-AUC_plasma_ ratios were published for dalbavancin. The concentrations of vancomycin were obtained in bone samples from 14 patients undergoing total hip arthroplasty after receiving an i.v. dose of 15 mg/kg of body weight (maximum, 1 g) vancomycin (20). Mean cortical and cancellous bone-to-serum ratios at the time of bone resection were 0.07 and 0.13, respectively. Another study was conducted with vancomycin administered to 30 patients undergoing a primary knee replacement (18 patients at a single i.v. dose of 500 mg; 12 patients at a single i.v. dose of 1,000 mg) and 8 patients undergoing revision knee replacement (at a single i.v. dose of 1,000 mg), none of which were due to infection. The overall mean synovial fluid-to-serum ratio at the time of surgery was 0.35 (21). Data were also published for linezolid: 10 patients undergoing primary total knee replacement received oral doses of 600 mg linezolid (q12h) over the 48 h before the surgery and an i.v. dose of 600 mg 1 h before induction of anesthesia. The overall mean bone and synovial fluid-to-serum ratios at the time of surgery were 0.40 and 0.92, respectively (22). Another study was performed in 18 patients undergoing elective total hip replacement and receiving a single i.v. dose of 1 g ertapenem. Median bone tissue- or synovial fluid-to-serum ratios were 0.13, 0.19, and 0.41 for cortical bone, cancellous bone, and synovial fluid, respectively (23). Additionally, a study was performed in 16 patients undergoing knee or hip replacement and receiving a single i.v. dose of 8 mg/kg daptomycin prior to surgery. The overall mean bone- and synovial fluid-to-plasma ratios at the time of surgery were 0.14 and 0.54, respectively (24). Finally, bone samples were collected in 30 patients receiving a single i.v. infusion of 1,000 mg dalbavancin at 0.5, 1, 3, 7, 10, or 14 days before undergoing elective orthopedic surgery. The mean bone-to-plasma AUC ratio was 0.13 (25).

All these data described in the literature show lower cortical bone, cancellous bone, and synovial fluid drug penetration for the drugs tested, with the exception of linezolid, compared to the data obtained for afabicin desphosphono when the distribution was evaluated in noninfected patients.

There has been a general recognition that assessing the unbound concentrations of antibiotics in the bone tissues would be advantageous in the interpretation of the concentrations required in bone for efficacy, with the bioactive drug being believed to be only the unbound drug at the site of action. Attempts to investigate the unbound concentrations of antibiotics in the bone tissues have been made by several researchers through the use of techniques such as *in situ* microdialysis (1, 26, 27). The latter technique was attempted for afabicin desphosphono, but *in vitro* experiments showed that it could not be applied because of its adsorption and solubility properties; therefore, in this study, as in the example studies cited above, the bone-to-plasma ratios are based on total drug concentrations determined in plasma, tissues homogenates, and fluids.

Nevertheless, the free fraction of afabicin desphosphono has been determined to be 2% in human plasma (protein binding estimate of 98% by ultrafiltration/equilibrium dialysis), and it was possible to determine it in synovial fluid as well. The mean value was 9.4% in synovial fluid (range, 2.4% to 34%). Consequently, the synovial fluid-to-plasma ratio was higher when considering the free bioactive fraction (mean free drug AUC ratio, 2.88; range, 0.75 to 10.4). Ratios of the free drug AUC in bone tissues to the free drug AUC in plasma obtained by microdialysis reported for vancomycin (28), daptomycin (29), and linezolid (30) were 0.8, 1.17, and 1.09, respectively. Comparison with these data suggests a great potential of afabicin desphosphono to have a high level of activity in bone tissues, even when taking into account the variable range of its free fraction in synovial fluid.

While the PK/pharmacodynamic (PD) parameter most likely to predict the efficacy of afabicin desphosphono is the free drug AUC/MIC based on mouse thigh infection models (31), the free afabicin desphosphono synovial fluid concentrations (minimum, 0.047 µg/ml; maximum, 0.149 µg/ml) above the MIC_90_ (0.015 µg/ml) of S. aureus observed over the complete dosing interval in this study are promising for the treatment of osteomyelitis.

Limitations to the study were the small number of patients at each sampling point and the fact that the data generated in this study may not reflect drug penetration in pathological situations. The concentrations in noninfected tissues were obtained, and the concentrations in infected bone and potentially in medullary bone may be higher than those measured in these otherwise healthy patients (1). Indeed, it was shown in animal models that the penetration of afabicin desphosphono was highly improved when the bone tissues were infected (12). Limitations were also related to the tissue processing method: as the tissues were homogenized, the concentrations represent a mix of intra- and extracellular concentrations. The possibility of some blood contamination also cannot be excluded.

In conclusion, data from this study show the high and sustained penetration of the afabicin active moiety in noninfected bone tissues of patients undergoing hip replacement surgery following dosing of 240 mg afabicin every 12 h. Afabicin was generally well tolerated without significant treatment-limiting adverse events. It has potential advantages over broad-spectrum antibiotics, namely, a narrow spectrum for more targeted therapy; a lower risk of colonization or infection with pathogenic gastrointestinal pathogens, such as C. difficile; and a mechanism of action that is unlikely to induce bacterial cross-resistance. These factors, along with its availability as both i.v. and oral formulations and its efficacy in animal models of osteomyelitis, strongly support the promise that afabicin may qualify as a reasonable choice for the treatment of staphylococcal bone and joint infections.

## MATERIALS AND METHODS

### Overall study design.

This was a multicenter, open-label study to evaluate the penetration of afabicin into bone in patients undergoing elective hip replacement surgery (study Debio 1450-108, ClinicalTrials.gov identifier NCT02726438). Seventeen patients were enrolled and randomized to a group receiving afabicin (*n* = 15) and a group not receiving afabicin (*n* = 2). The study population consisted of male and female patients 18 to 75 years of age (inclusive) with a body mass index (BMI) of 18 to 35 kg/m^2^ (inclusive) and a weight of at least 50 kg.

Patients received 3 oral administrations of afabicin (as the bis-ethanolamine salt in 5% dextrose solution) at a dose of 240 mg approximately 12 h apart, without regard to food intake. They were randomized into 4 groups, with the last dose of afabicin being given approximately 2, 4, 6, or 12 h prior to the scheduled surgery time. If the surgery was delayed by more than 12 h postdose, the patients could receive up to 2 additional administrations (approximately 12 h apart) to ensure that the last dose was administered between 2 and 12 h prior to surgery and that steady-state conditions were maintained. Tissues from the 2 patients who did not receive afabicin were collected for bioanalytical method validation. Afabicin was administered as an adjunct to the scheduled surgery and did not replace standard-of-care antibiotics. The study was approved by the following institutional review boards (IRBs): Schulman Associates IRB, Durham, NC; SHARP Center for Research IRB, San Diego, CA; and BRANY, Lake Success, NY.

### Pharmacokinetic sample collection and preparation.

Blood samples were collected in K_2_EDTA tubes before the last dose; at 0.5, 1, 2, 3, 5, 8, and 12 h after the last dose; and at the time of bone resection. The samples were centrifuged at 2,500 × g for 10 min at 4°C within 30 min of collection, and the plasma was stored at approximately −70°C. During surgery, synovial fluid was collected from the joint with a syringe and stored in K_2_EDTA tubes at −70°C. Cortical and cancellous bones, bone marrow, and soft tissue samples were collected during surgery, and aliquots were stored at −70°C. Blood, cancellous and cortical bones, bone marrow, soft tissue, and synovial fluid samples were protected from sun and artificial light exposure during collection and processing. The time of resection, defined as the time when the bone was disconnected from the local blood flow, and the exact time between the last dose of afabicin and the surgery were recorded.

### Bioanalysis.

Bioanalytical methods were used according to the bioanalytical laboratory’s standard operating procedures (Atlanbio, St-Nazaire, France) and EMA guidance (32). A full validation of a sensitive assay for afabicin and/or afabicin desphosphono in plasma and cortical bones, including the precision, accuracy, reproducibility, limit of quantitation, recovery, and selectivity, was completed and approved prior to sample analysis.

Plasma and synovial fluid samples were analyzed using acetonitrile protein precipitation followed by reverse-phase high-performance liquid chromatography and detection by triple-quadrupole mass spectrometry with heated electrospray ionization in the positive mode (TSQ Quantum Ultra; Thermo Fisher Scientific). The calibration curve ranged from 5 to 5,000 ng afabicin and afabicin desphosphono/ml plasma. Synovial fluid samples were analyzed after 1/5 dilution in human plasma. The accuracy was between 94.4 and 99.6% and 92.6% and 96% for afabicin and afabicin desphosphono, respectively, and the imprecision (CV) was 1.1 to 4.8% and 1.2 to 2.9% for afabicin and afabicin desphosphono, respectively. Cortical bone, cancellous bone, bone marrow, and soft tissue samples were homogenized using a cryogenic mill SPEX 6870 freezer/mill. Bone, bone marrow, or soft tissue homogenates (10 mg ± 1 mg) were weighed and extracted using liquid-liquid extraction with methyl *tert*-butyl ether, followed by reverse-phase high-performance liquid chromatography and triple-quadrupole mass spectrometry with heated electrospray ionization in positive mode (TSQ Quantum Ultra; Thermo Fisher Scientific). Calibration curves in cortical bone ranged from 0.01 to 10 ng afabicin desphosphono/mg tissue. The method was validated according to the EMA guidance on bioanalytical method validation (32). The accuracy was 91.7% to 102%, and the imprecision (CV) was ≤15.8%. The method was cross-validated with human cancellous bone, bone marrow, and soft tissue in order to demonstrate that the various human bone tissue samples could be analyzed with calibration standards prepared in human cortical bone and quality control samples prepared in a corresponding matrix. Bone samples from the 2 patients who did not receive afabicin were used as blank matrices for calibration and quality control samples.

### Determination of unbound fraction in plasma and synovial fluid.

The protein binding of afabicin desphosphono in plasma and synovial fluid was determined in the study samples and blank matrices (obtained from different suppliers: Transi-Hit Bio, Articular Engineering, Cambridge Bioscience Limited UK, and Seralab) spiked with the compound by rapid equilibrium dialysis (RED) (33). Three hundred microliters of dialysis buffer (RED phosphate buffer, pH 7.2; Thermo Fisher Scientific) were added to the dedicated RED plate buffer chambers (Thermo Fisher Scientific), and 100 µl of plasma or synovial fluid adjusted to pH 7.4 was aliquoted in triplicate into the dedicated RED plate sample chambers. The RED plate was then sealed with sealing tape and incubated at 37°C in a thermomixer at 350 rpm for 5 h. After a 5-h incubation, 50 µl of each postdialysis sample was diluted in plasma before extraction by acetonitrile protein precipitation. Samples were then analyzed by liquid chromatography coupled to tandem mass spectrometry (Waters Xevo TQS) in electrospray positive mode with a calibration curve prepared in human plasma.

### Pharmacokinetic analyses.

The area under the curve over the dosing interval (12 h) at steady state (AUC_τ_), the maximum observed plasma concentration (*C*_max_), the measured concentration at the beginning and end (12 h) of the third dosing interval (*C*_trough_ and *C*_12_, respectively), and the half-life of afabicin desphosphono were calculated by noncompartmental analysis (NCA) using Phoenix WinNonlin (v6.3) software (Certara L.A.). The NCA was performed from the individual full plasma PK profile for each patient using actual sampling times. For the tissues, synovial fluid, as well as plasma collected at the time of bone resection, the NCA was performed from the composite profile constructed from the mean of the individual concentrations available at each nominal resection time point. The density of all biological matrices was considered 1 for calculation of the ratios. The mean and coefficient of variation (CV) data presented in this article for afabicin desphosphono are geometric means and CVs. The concentrations and PK parameters are based on total afabicin desphosphono unless specified otherwise (e.g., AUC is total AUC and the free drug AUC is the AUC of unbound compound).

## Supplementary Material

Supplemental file 1
