# Supplementary material for: Bone and Joint Tissue Penetration of the Staphylococcus-Selective Antibiotic Afabicin in Patients Undergoing Elective Hip Replacement Surgery
Source: Antimicrob Agents Chemother. 2019 Feb 26;63(3):e01669-18. doi: 10.1128/AAC.01669-18 (PMC6395911; doi:10.1128/AAC.01669-18)
Supplement: Supplemental file 1 [file AAC.01669-18-s0001.pdf]

Table S1: Individual replicates and median concentrations of afabicin desphosphono in cortical bone, cancellous bone, bone marrow and soft tissue collected at time of resection.

Subject	Compartment	Aliquot	Concentration (ng/g)		Median concentration (ng/g)
			Replicate 1	Replicate 2	
01	Cortical bone	1a	201	239	**224**
		1b	314	346	
		2	178	208	
01	Cancellous bone	1a	478	577	**497**
		1b	468	545	
		2	515	295	
01	Bone marrow	1a	401	472	**363**
		1b	345	365	
		2	361	307	
01	Soft tissue	1a	445	364	**473**
		1b	540	500	
		2	505	420	
02	Cortical bone	1a	472	471	**472**
		1b	478	491	
		2	419	471	
02	Cancellous bone	1a	822	848	**739**
		1b	497	446	
		2	740	737	
02	Bone marrow	1a	527	563	**515**
		1b	502	483	
		2	566	500	
02	Soft tissue	1a	625	674	**617**
		1b	699	571	
		2	573	609	
03	Cortical bone	1a	504	422	**458**
		1b	518	493	
		2	270	178	
03	Cancellous bone	1a	477	596	**590**
		1b	349	692	
		2	713	583	
03	Bone marrow	1a	745	637	**609**
		1b	594	623	
		2	588	347	
03	Soft tissue	1a	498	436	**467**
		1b	639	673	
		2	398	353	
04	Cortical bone	1	730	635	**683**
04	Cancellous bone	1	1060	1240	**1020**
		2	984	892	
04	Bone marrow	1	612	825	**825**
		2	932	-[a]	
04	Soft tissue	1	477	436	**457**
05	Cortical bone	1	590	571	**581**
05	Cancellous bone	1	953	1080	**1020**

Subject	Compartment	Aliquot	Concentration (ng/g)		Median concentration (ng/g)
			Replicate 1	Replicate 2	
05	Bone marrow	1	875	853	**864**
05	Soft tissue	1	640	670	**655**
06	Cortical bone	1	176	183	**180**
06	Cancellous bone	1a	601	264	**599**
		1b	623	597	
06	Bone marrow	1	236	299	**268**
06	Soft tissue	1	296	370	**333**
07	Cortical bone	1	191	196	**283**
		2	370	378	
07	Cancellous bone	1	434	435	**435**
07	Bone marrow	1	319	309	**314**
07	Soft tissue	1	455	416	**436**
08	Cortical bone	NA			**NA**
08	Cancellous bone	NA			**NA**
08	Bone marrow	NA			**NA**
08	Soft tissue	NA			**NA**
09	Cortical bone	1	333	413	**373**
09	Cancellous bone	1a	368	3530	**505**
		1b	442	567	
09	Bone marrow	1	485	484	**485**
09	Soft tissue	1	357	376	**367**
10	Cortical bone	1	348	327	**338**
		2	NA	NA	
10	Cancellous bone	1a	1620	743	**913**
		1b	964	862	
10	Bone marrow	1	778	751	**765**
10	Soft tissue	1	724	814	**769**
11	Cortical bone	1	197	193	**195**
		2	198	190	
11	Cancellous bone	1	462	444	**453**
11	Bone marrow	1	500	497	**499**
11	Soft tissue	1	410	540	**475**
		1b	-[a]	-[a]	
12	Cortical bone	1	533	543	**538**
12	Cancellous bone	1	867	899	**883**
12	Bone marrow	1	845	858	**852**
12	Soft tissue	1	913	930	**922**
13	Cortical bone	NA			**NA**
13	Cancellous bone	NA			**NA**

Subject	Compartment	Aliquot	Concentration (ng/g)		Median concentration (ng/g)
			Replicate 1	Replicate 2	
13	Bone marrow	1	1180	-[a]	**1180**
13	Soft tissue	1	1060	1040	**1050**
14	Cortical bone	1	152	143	**148**
14	Cancellous bone	1	516	466	**468**
		2	430	469	
14	Bone marrow	1	359	383	**363**
		2	367	344	
14	Soft tissue	1	343	278	**303**
		2	274	328	

NA: no sample available; 1a and 1b: same aliquot (1) analysed in separate batches; [a] No sufficient tissue for two replicates for reassay.

Following analysis of replicates for the two aliquots of the first patients, the differences between some of the replicates was higher than 25% in some cases. Consequently, aliquot 1 was analysed in duplicates for all patients in one run; if the difference between the duplicates (100 • [Replicate 1 – Replicate 2 / mean]) was higher than 25%, a reanalysis was conducted in duplicates. In addition, if the mean of the two replicates was higher than 50% of the median value of all tissues, reanalysis of the aliquot 1 and analysis of aliquot 2 were performed in duplicates. These repeat analyses were done to increase the accuracy of tissue concentration data.

The median of all the replicates is reported as recommended by the Global Bioanalysis Consortium Harmonization Team [1].

Reference

1 **Fluhler E, Vazvaei F, Singhal P, Vinck P, Li W, Bhatt J, de Boer T, Chaudhary A, Tangiuchi M, Rezende V, Zhong D. 2014.** Repeat Analysis and Incurred Sample Reanalysis: Recommendation for Best Practices and Harmonization from the Global Bioanalysis Consortium Harmonization Team. AAPS J. 16(6):1167-1174.

.
